# Olfactory Cues in Infant Feeds: Volatile Profiles of Different Milks Fed to Preterm Infants

**DOI:** 10.3389/fnut.2020.603090

**Published:** 2021-01-15

**Authors:** Mariana Muelbert, Frank H. Bloomfield, Shikha Pundir, Jane E. Harding, Chris Pook

**Affiliations:** Liggins Institute, University of Auckland, Auckland, New Zealand

**Keywords:** breastmilk, neonatal nutrition, volatile compounds, smell, preterm (birth), infant formula, SPME-GC/MS

## Abstract

**Background:** Smell is determined by odor-active volatile compounds that bind to specific olfactory receptors, allowing us to discriminate different smells. Olfactory stimulation may assist with digestion and metabolism of feeds in the neonate by activation of the cephalic phase response of digestion. Infants' physiological responses to the smell of different milks suggest they can distinguish between breastmilk and infant formula. We aimed to describe the profile of volatile compounds in preterm breastmilk and investigate how this differed from that of other preterm infant feeding options including pasteurized donor breastmilk, breastmilk with bovine milk-based fortifier, human milk-based products and various infant formulas.

**Methods:** Forty-seven milk samples (13 different infant formulas and 34 human milk-based samples) were analyzed. Volatile compounds were extracted using Solid Phase Micro Extraction. Identification and relative quantification were carried out by Gas Chromatography with Mass Spectrometry. Principal Component Analysis (PCA) and one-way Analysis of Variance (ANOVA) with Tukey's HSD (parametric data) or Conover's *post-hoc* test (non-parametric data) were used as appropriate to explore differences in volatile profiles among milk types.

**Results:** In total, 122 compounds were identified. Breastmilk containing bovine milk-based fortifier presented the highest number of compounds (109) and liquid formula the lowest (70). The profile of volatile compounds varied with 51 compounds significantly different (adjusted *p* < 0.001) among milk types. PCA explained 47% of variability. Compared to preterm breastmilk, the profile of volatile compounds in breastmilk with added bovine milk-based fortifier was marked by presence of fatty acids and their esters, ketones and aldehydes; infant formulas were characterized by alkyls, aldehydes and furans, and human milk-based products presented high concentrations of aromatic hydrocarbons, terpenoids and specific fatty acids.

**Conclusions:** Sensory-active products of fatty acid oxidation are the major contributors to olfactory cues in infant feeds. Analysis of volatile compounds might be useful for monitoring quality of milk and detection of oxidation products and environmental contaminants. Further research is needed to determine whether these different volatile compounds have biological or physiological effects in nutrition of preterm infants.

## Introduction

Smell and taste are critical to food appreciation and are determined, in part, by volatile substances called odorants. Odorants are small, organic and inorganic volatile molecules which bind to olfactory receptors, triggering electrical impulses in the olfactory centre. Odorants are produced in a variety of metabolic pathways by all living organisms. Examples include microbial fermentation or endogenous enzymatic activity (1).

The activation of specific olfactory receptors allows the brain to discriminate smells (2). Perception of odorants in humans is estimated by the odor activity value (OAV), which takes into account the ratio of the concentration of an individual substance in a sample and the threshold concentration of this substance in the respective matrix (minimal concentration) that can be detected by the human olfactory receptors. Not all volatile compounds are sensory active as compounds with OAV < 1 cannot be perceived by the human nose (3). However, it has been suggested that newborn infants might be able to detect odors at lower threshold concentrations than adults (4).

Olfactory and gustatory stimulation may be important for digestion and metabolism of feeds in the neonate by triggering reflexes in the brain that promote salivation, peristaltic movements and release of hormones and enzymes related to digestion, thereby initiating the digestive process even before food reaches the stomach (5). These reflexes are referred to as the cephalic phase response (CPR). Observations of infants' physiological responses to breastmilk and infant formula demonstrate that they are capable of distinguishing between the smell of different milks, indicating that odorants present in breastmilk differ from those in infant formula (6–8). Exposure to odor of breastmilk has been related to improved response to pain and enhanced sucking skills (7, 9–11), with babies exposed to the smell of breastmilk displaying more non-nutritive sucking bouts (7) and longer sucking bouts leading to greater milk consumption (11) compared to babies exposed to smell of formula or water, respectively. Furthermore, recent evidence indicates that smell and taste of milk may contribute to early attainment of full oral feeds and shorter hospital stay, although the quality of evidence is low (12, 13). Yet, sensory stimulation is often under-appreciated in the care of preterm infants, despite functional olfactory receptors being present from 28 weeks' gestation onwards (14).

Research into human milk composition has demonstrated that breastmilk metabolic profile (i.e., concentration of different amino acids, fatty acids, sugars, etc.) is influenced by gestational age at birth and postnatal age (15–17). In addition, maternal dietary intake contributes to flavor and nutritional composition of breastmilk (18–20), suggesting that the components in breastmilk responsible for flavor are not fixed. Positive correlations have been identified between macronutrient composition and breastmilk flavor, with fat and protein content correlated with perceived creamy flavor, carbohydrate content with sweet flavor and bitterness of maternal diet with perceived bitter flavor (21). Thus, it has been suggested that, prior to weaning, formula fed infants are exposed to fewer flavors compared to breastfed infants (22, 23).

Different volatile compounds in infant feeds may contribute to feed tolerance and digestion, both through palatability and activation of the CPR. This could be important in preterm babies, in whom establishment of full enteral feeding may be delayed and who may not receive olfactory stimulation when milk feeds are given via gastric tube. Previous studies using a variety of techniques for extraction of volatile compounds [such as purge-trap (24), solvent-assisted extraction (25), solid-phase micro extraction (SPME) (26) and electronic nose (27)] have reported that aldehydes, ketones, fatty acids, alcohols and terpenoids are volatile compounds present in term breastmilk (24, 25) and infant formulas (24, 26, 27). However, it remains unclear which volatile compounds in preterm breastmilk, breastmilk fortifiers and substitutes for mother's own milk that commonly are used for feeding preterm babies may contribute to olfactory stimulation. Therefore, the purpose of this study was to analyze the volatile compounds in a variety of different milks routinely fed to preterm infants, including expressed breastmilk, fortified breastmilk, pasteurized breastmilk, human milk-derived commercial products and several infant formulas. Volatile compounds in milk were extracted without solvent at the temperature at which they are fed to the infant, rather than at higher temperatures, in order to provide a better understanding of the compounds that might be biologically relevant and, perhaps, play a physiological role in olfactory perception in the newborn.

## Materials and Methods

### Samples

#### Human Milk Samples

##### Preterm Breastmilk

Preterm breastmilk (PBM) samples were obtained from 15 mothers of participants in an ongoing randomized control trial investigating different nutritional support strategies for moderate-to-late preterm infant admitted to Neonatal Intensive Care Units (NICUs) in the city of Auckland, New Zealand (DIAMOND trial; ACTRN12616001199404). This study was approved by New Zealand's Health and Disability Ethics Committee (HDEC 16/NTA/90 and 18/CEN/256) and participants provided written informed consent. Information on the collection protocol can be found elsewhere (28). Briefly, samples were collected in the first 10 days after birth and mothers were requested to express milk from their right breast using an electronic breast-pump (Medela Symphony®, Switzerland) into disposable sterile bottles (Medela®) at least 2–3 h after the previous milk expression. After the right breast was completely emptied, the total volume of expressed breastmilk was vortexed for 2 min at high speed to ensure homogeneity and 2 mL of breastmilk was collected using a sterile enteral syringe (BD, Singapore) and aliquoted in equal amounts into four low protein binding microtubes (Eppendorf, Germany), one aliquot of which was used in this study. After aliquoting, samples were frozen at −80°C and stored for 12–15 months until analysis.

##### Pasteurized Breastmilk

Pasteurized donor breastmilk (PDBM) was obtained from four different mothers who donated breastmilk to the Human Milk Bank in Christchurch, New Zealand. The milk bank pools breastmilk from single donors to make up batches of milk for pasteurization. More information about the Human Milk Bank protocols can be found elsewhere (29). No maternal or infant information was available under the ethical approval for research involving these samples. De-identified samples were shipped frozen from the Human Milk Bank to our facility and stored at −20°C freezer until analysis, which took place within 6 months of pasteurization.

##### Human Milk-Based Formulas

Samples of three human milk-based products were included. Two of the products are ready-to-feed (RTF) formulas designed to contain protein, fat and calories from pasteurized donor human milk with essential minerals added to deliver standardized caloric and micronutrient content. Prolact RTF 24 and Prolact RTF 28 (Prolacta Bioscience, Inc., California, USA) provide 40.5 calories and 1.1 g protein and 47.5 calories and 1.6 g protein per 50 mL, respectively. Additionally, we included samples of PremieLact (Prolacta Bioscience), nutritionally incomplete product designed for trophic feeding only and providing 0.7 calories per mL with no minerals added. Products were shipped frozen to our facility and stored for 4 months at −20°C until analysis.

#### Fortified Breastmilk

##### Human Milk-Based Fortifier

Two human milk-based fortifiers (HMF) were added to pasteurized breastmilk from a single donor and prepared according to manufacturer's instructions. Human milk-based fortifiers are made from pasteurized breastmilk with minerals added. Products (Prolact Plus 4 H^2^MF and Prolact Plus 6 H^2^MF, Prolacta Bioscience) were shipped frozen to our facility and stored for 4 months at −20°C until analysis. Just prior to analysis, products were added to pasteurized breastmilk to provide ~41 calories and 1.2 g protein (Prolact Plus 4 H^2^MF) and 45 calories and 1.4 g protein (Prolact Plus 6 H^2^MF) per 50 mL.

##### Bovine Milk-Based Fortifier

Breastmilk containing bovine milk-based fortifier (BMF) was obtained from 10 mothers of preterm infants (<32 weeks' gestation or with birthweight < 1,800 g) admitted to the Auckland City Hospital NICU who had fortification of breastmilk with bovine milk-based multi-nutrient fortifier (PreNan, FM 85, Nestlé, Vevey, Switzerland) prescribed for clinical reasons. This product is a powder made of extensively hydrolyzed bovine milk protein, carbohydrates, lipids, trace elements and vitamins and fortification was done following manufacturer's instructions. This study was approved by New Zealand's Health and Disability Ethics Committee (HDEC 18/CEN/256) and participants provided written informed consent. No maternal or infant information was collected. Fortified breastmilk samples that had been stored at 4°C for >8 h, the limit for clinical use in this hospital but still within the timeframe used by many nurseries (30), were collected between March and July 2019. Samples were de-identified and vortexed for 30 s at medium speed before a 2 mL volume was collected and stored at −80°C for 6 months until analysis.

#### Infant Formulas

##### Liquid Formulas

Six liquid infant formulas (LF) were included in the analysis: two preterm infant formulas (Aptamil Preterm Gold Plus, Danone Nutricia NZ Ltd, Auckland, New Zealand; Pre Nan Gold, Nestlé); one low-birth weight infant formula (S-26 LBW, Wyeth Nutritionals Singapore Pte Ltd, Singapore), and three term infant formulas (S26-Gold, Wyeth Nutritionals, Askeaton, Ireland; Aptamil Gold Plus, Danone Nutricia NZ Ltd; Similac Advance Pro, Abbot Laboratories, Columbus, Ohio, USA). All liquid formulas contained cows' milk protein and mixed vegetable oils containing long- and medium-chain polyunsaturated fatty acids. All samples were unopened prior to analysis and analyzed within shelf-life stated in the product.

##### Powder Formulas

We analyzed seven stage 1 (from birth to 12 months) powder infant formulas (PF) with different sources of protein: 4 cows' milk formulas (Similac Pro-Advance, Abbot Laboratories; SMA, Wyeth Nutritionals Singapore Pte Ltd; Nurture, Heinz Wattie's, Hastings, New Zealand; S-26 Gold, Wyeth Nutritionals Singapore Pte Ltd); one goat milk formula (Karicare Goats' Milk, Danone Nutricia NZ Ltd); one soy-based formula (Kericare+ Soy Milk, Danone Nutricia NZ Ltd) and one formula made of cow's milk with only A2 β-casein protein (S-26 A2 Milk, Wyeth Nutritionals Singapore Pte Ltd). All formulas were prepared using boiled tap water on the day of analysis following manufacturers' instructions and analyzed within shelf-life stated in the product.

### Sample Preparation

To minimize potential batch effects, samples were randomly allocated into batches for analysis. Frozen samples were thawed at 4°C for 4 h. Samples were vortexed at low speed to ensure homogeneity and 400 μL of milk was transferred into 10 mL headspace amber vials with magnetic screw caps and polytetrafluoroethylene-lined, silicone septa (Thermo Fisher Scientific Inc., New Zealand). The same volume of milk was analyzed for all samples to ensure standardization and comparison among different milk types.

### Quality Control

Procedural blanks contained 400 μL of the same boiled water used to prepare the powder infant formulas. Two types of Quality Controls (QC) were prepared: one contained a pool of all preterm and pasteurized breastmilk samples (PBM and PDBM) and another contained a pool of powder and liquid formula samples (LF and PF). Bovine milk-based fortified breastmilk (BMF) and human milk-based products were not included in QCs due to the limited volume available. Each type of QC was aliquoted into three vials each containing 400 μL. The procedural blanks and the human and formula milk QCs were placed at the beginning, middle and end of each batch. All samples, QCs and blanks were spiked with 10 μL of ultrapure water containing 2-chlorophenol at 20 μL.L^−1^ as an internal standard (IS) (Sigma-Aldrich, Sidney, Australia). Data from the QC analyzes were processed in parallel with the samples to confirm reproducibility of retention times and peak areas among batches.

### Analysis of Volatile Compounds

Analysis of volatile compounds was performed by headspace solid phase micro extraction gas chromatography with mass spectrometry (SPME-GC-MS). A Gerstel MPS2 autosampler was used to equilibrate samples at 37°C, with continuous stirring, for 10 min. The SPME fiber used for extraction of volatile compounds was divinylbenzene-carboxen-polydimethylsiloxane (DVB-CAR-PDMS) 50/30 μm (Supelco) measuring 20 mm in length. A 10 mL amber vial was used to prevent contact between the milk and the SPME fiber. Extraction of volatile compounds occurred for 10 min. The GC-MS instrument was a Thermo Fisher Trace GC Ultra with a Programmable Temperature Vaporiser connected to a Thermo ISQ mass spectrometer. The carrier gas was zero grade helium (99.995%, BOC New Zealand) at a constant flow rate of 1.1 mL per minute. Upon injection the SPME fiber was simultaneously desorbed and conditioned in the GC injector in high pressure splitless mode using a low-volume SPME-specific deactivated liner (0.75 mm ID) at 250°C for 10 min. The column was a Phenomenex 1701 capillary column (30 m × 250 μm × 0.25 μm). Oven temperature was set at initial temperature of 35°C and held for 4 min, followed by an increase of 5°C/min up to 165°C followed by an increase of 50°C/min up to 265°C. Data were acquired at a scan rate of 5 Hz in the range m/z 20–300.

### Compound Identification and Statistical Analysis

Deconvolution and identification of features in the GC-MS data was performed using Agilent MassHunter Unknowns software (Agilent technologies) searching the 2017 version of the National Institute of Standards & Technology mass spectral library (NIST, USA) using retention time calibration by Kovats Index and a match factor threshold of 80%. Identities were filtered to exclude features with a signal-to-noise ratio < 10 or that were identified in less than two samples (at least one sample and its QC). Authentic standards were run to verify the identities of short- and medium-chain fatty acids and their esters, as well as various alcohols, aldehydes and ketones for which standards were readily available. Compounds identified with authentic standards are indicated in Supplementary Table 1. These annotations comply with criteria for level 1 metabolite identification described by the Metabolomics Standards Initiative (MSI) (22). All other annotations complied with level 2 metabolite identification. Automated integration of extracted-ion chromatogram (EIC) signals was carried out using MassHunter Quantitative Analysis software (Version 10.0; Agilent Technologies), with visual inspection and manual correction where necessary. Peak areas were normalized to the internal standard (2-chlorophenol) and subtracted from procedural blanks (boiled water). Unsupervised multivariate test (Principal Component Analysis, PCA) and one-way Analysis of Variance (ANOVA) with Tukey's HSD *post-hoc* were used to explore differences between volatile profiles among milk types. When assumptions for ANOVA were not met, pairwise multiple comparisons were analyzed by non-parametric test (Kruskal-Wallis with Conover's *post-hoc* test). To account for multiple comparisons, false discovery rate (FDR = 0.05, Benjamini-Hochberg) adjusted *p* value of <0.05 was considered statistically significant. Relative abundance of identified features are presented as mean peak area and standard deviation. In order to improve visualization and comparison of PCA and heatmap data, each compound's relative peak area was normalized (mean divided by standard deviation of each compound) and transformed using generalized logarithmic transformation. Statistical analysis was performed with *statsmodels, scipy* and *scikit-posthocs* packages in Python 3.6.5 (Anaconda 3 v5.2, Continuum Analytics).

## Results

### Volatile Profile of Included Milk Types

In total, 47 samples (13 infant formulas and 34 human milk-based samples) were analyzed. Based on Kovats Retention Index and similarity with library spectra or confirmation with authentic standards, identities were assigned to 121 features: 24 alkyls; 17 fatty acids (FA) and FA esters; 14 siloxanes; 12 aldehydes; 12 ketones; 12 aromatic hydrocarbons; eight terpenoids; six alcohols; four furans; three chlorination by-products; two sulfur compounds; one ether; one amide; one hydrocarbon derivate; one phenolic compound; one acrylate; one microbial metabolite (indole), and one pharmaceutical compound (chlorobutanol). One unknown feature was detected to which no identity could be robustly assigned (Supplementary Table 1 – Relative peak area of volatile compounds in different milk types). Where information is available, odor threshold, odor activity value (OAV) and odor description for compounds are presented in Supplementary Table 2.

**Figure 1:**
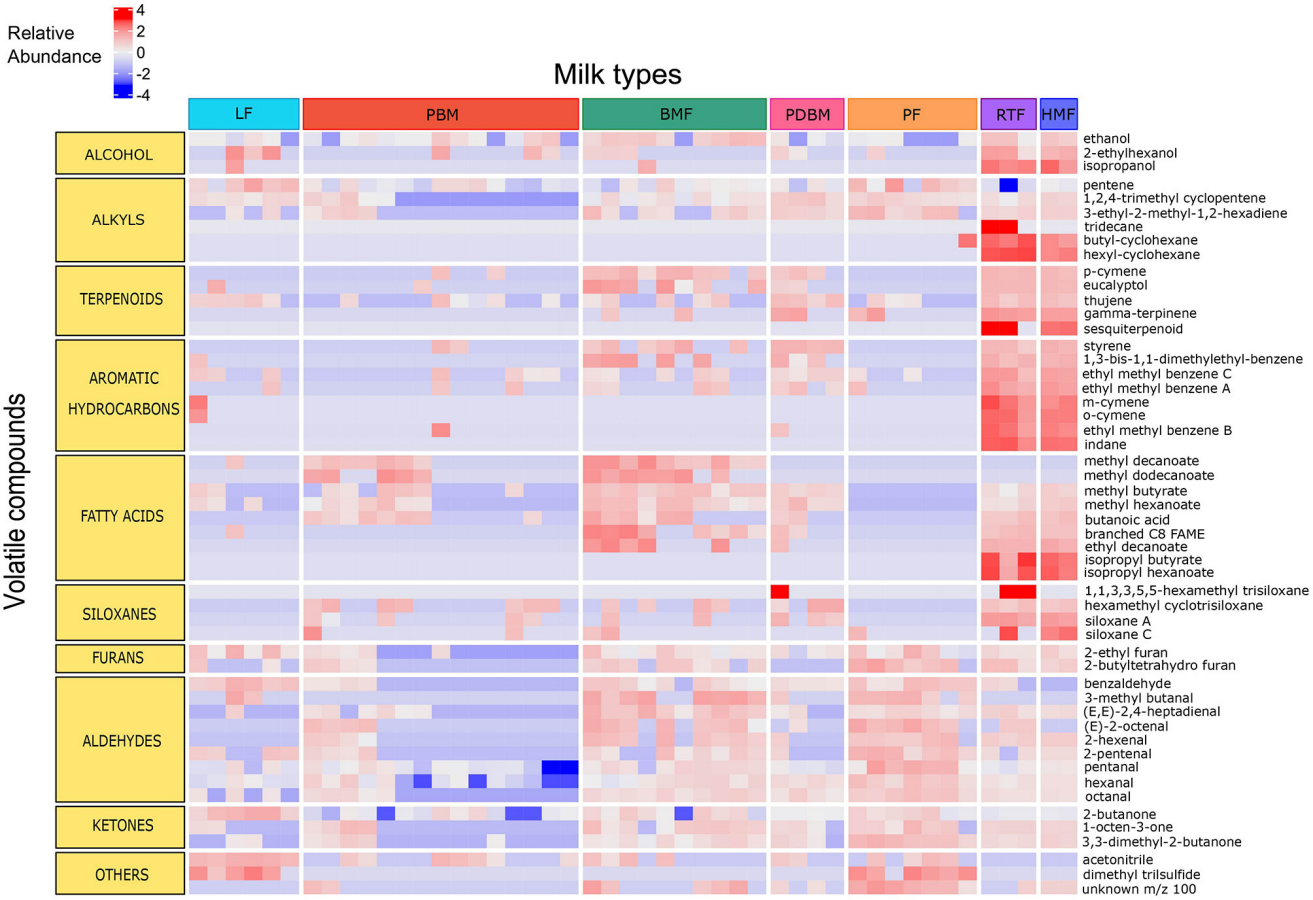
Relative abundance of compounds significantly different across milk types. Compounds represented in rows and samples represented in columns. Data are log-transformed and color scale indicates intensity of relative abundance of a compound. Positive values (red) represent high abundance, negative values (blue) represent low abundance, and zero represents moderate abundance. LF, Liquid Formula; PBM, Preterm breastmilk; BMF, Bovine milk-based fortified breastmilk; PDBM, Pasteurized donor breastmilk; PF, Powder Formula; RTF, Human milk-based Ready-To-Feed formula; HMF, Human-milk based fortified breastmilk.

Breastmilk containing bovine-based fortifier presented the highest number of compounds (109), followed by human milk-based ready-to-feed formula (94), preterm breastmilk (91), pasteurized breastmilk (83), and breastmilk containing human milk-based fortifier (81). Powder formula presented more compounds than liquid formula (88 and 70, respectively). Despite different sources of nutrients (soy, goat, and bovine milk), all powder formulas presented a similar profile of volatile compounds. The distribution of volatile compounds in each milk type is illustrated in Figure 1.

### Comparison Between Different Milk Types

**Figure 2:**
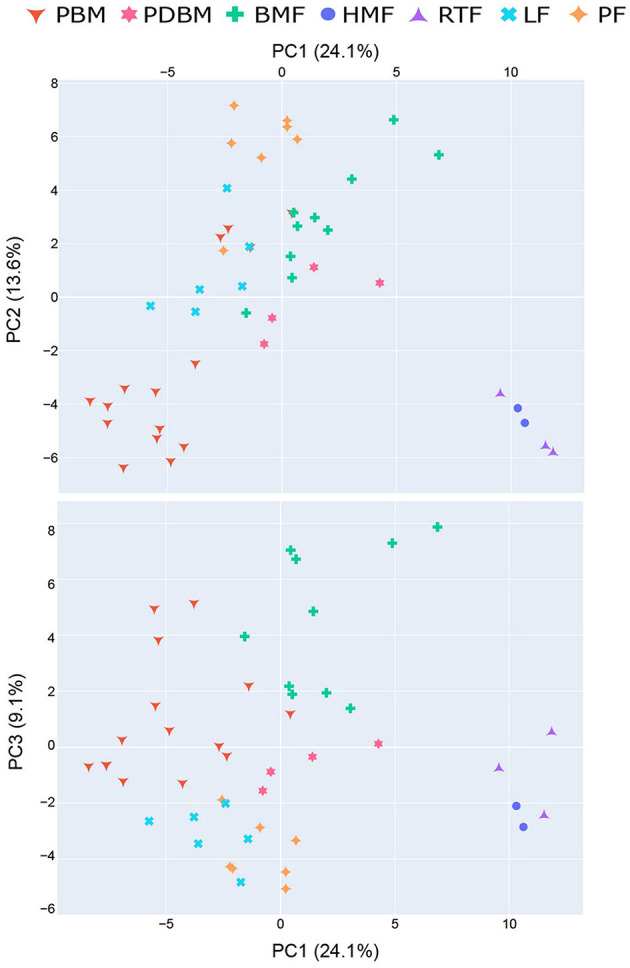
Principal Component Analysis (PCA) explained 47% of variability of different milk types in the first three components. HMF, Human-milk based fortified breastmilk; PBM, Preterm breastmilk; BMF, Bovine milk-based fortified breastmilk; RTF, Human milk-based Ready-To-Feed formula; PF, Powder Formula; LF, Liquid Formula; PDBM, Pasteurized Donor breastmilk.

The profile of volatile compounds varied significantly among the different milk types. Principal Component Analysis (PCA) explained 47% of the variance among volatile profiles in the first three components (PC1 24.1%; PC2 13.6% and PC3 9.1%) (Figure 2). Human-milk based products (HMF and RTF) were clearly differentiated from the other milk types, indicated by a separate cluster in PC1. The loadings for this component reveal that the compounds driving differences between these milk types were aromatic hydrocarbons and specific FA esters and terpenoids which were more abundant in HMF and RTF. Almost all preterm breastmilk (PBM) samples and human milk-based products (HMF and RTF) were separated from the other milk types in PC2, clustering in the lower portion of PC2 axis. The loadings show these differences result from elevated concentrations of aldehydes and ketones in infant formulas (LF and PF), bovine milk-based fortified breastmilk (BMF) and pasteurized breastmilk (PDBM) compared to PBM, HMF and RTF. Breastmilk samples (PBM, PDBM, and BMF) were differentiated from infant formulas by PC3, with the infant formulas forming a cluster low on PC3 axis. These differences were mostly due to decreased concentrations of FA and FA esters in the infant formula samples.

### *Post hoc* Group Comparisons

**Figure 3:**
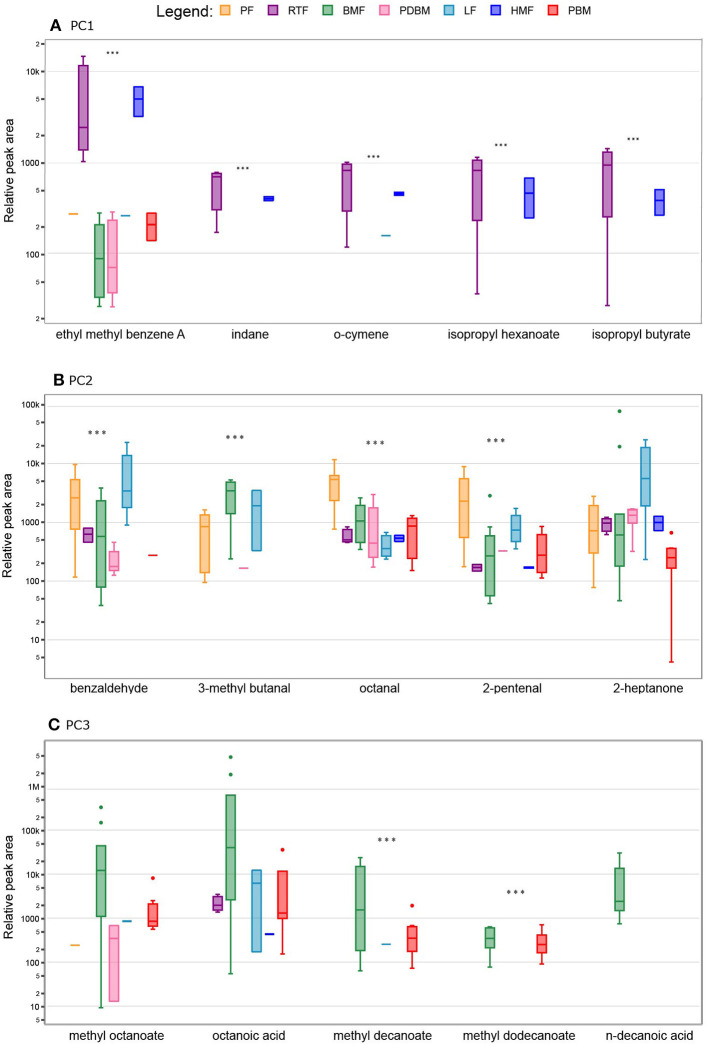
Relative peak area of the top five volatile compounds in different milk types contributing to Principal Component (PC) Analysis. Boxes represent median and interquartile range, whiskers represent highest and lowest peak area detected and dots represent outliers. Milk types are not shown when compound was not detected. **(A)** Aromatic hydrocarbons and specific fatty acids contributed to differences observed in PC1; **(B)** aldehydes and ketones contributed to differences observed in PC2; **(C)** fatty acids contributed to differences observed in PC3. PF, Powder Formula; RTF, Human milk-based Ready-To-Feed formula; BMF, Bovine milk-based fortifier; PDBM, Pasteurized donor breastmilk; LF, Liquid Formula; HMF, Human-milk based fortified breastmilk; PBM, Preterm breastmilk; *** *p* < 0.001.

The differences observed in the PCA were supported by *post hoc* group comparisons. The concentration of fifty-one compounds differed significantly (FDR adjusted *p* < 0.05) across the different milk types (Figure 1). Compared to the other milk types, significantly higher mean peak areas of the following were detected in the human milk-based products (HMF and RTF): aromatic hydrocarbons indane, styrene, *meta* and *ortho* cymene and isomeric forms of ethyl methyl benzene (*p* < 0.001); FA esters isopropyl butyrate and isopropyl hexanoate (*p* < 0.001), and the unknown sesquiterpenoid (*p* < 0.001). Compared to samples containing breastmilk, infant formulas presented higher abundance of aldehydes such as hexanal, octanal, pentanal, and benzaldehyde (*p* < 0.001), alkyls such as pentane and 3-ethyl-2-methyl-1,3-hexadiene (*p* < 0.001), ketones such as 2-butanone and pinacolone (*p* < 0.001) and furans such as 2-ethyl furan and 2-butyltetrahydro furan (*p* < 0.001). Samples of breastmilk with bovine-based fortifier presented increased abundance of FA such as butanoic acid (*p* < 0.01) and esters of FA such as methyl hexanoate, methyl butyrate and methyl decanoate (*p* < 0.001) compared to the other milk types. The main differences among milk types with respective pairwise comparisons are illustrated in Figure 3.

## Discussion

### Main Findings

The profile of volatile compounds is an important indicator of milk integrity (26, 31, 32) and may also contribute to the palatability and digestibility of feeds. Our results demonstrate that the volatile profile of preterm breastmilk is different from other infant feeding options including pasteurized breastmilk, breastmilk with bovine milk-based fortifier, products made from pasteurized breastmilk and various liquid and powder infant formulas, and that these different types also differ from each other. These clear distinctions amongst milks result mostly from the varying concentration of aldehydes, ketones, aromatic hydrocarbons, terpenoids, fatty acids, and fatty acids esters. Our results identified a more diverse profile of volatile compounds in breastmilk compared to infant formulas. In general, the profile of volatile compounds in breastmilk milk samples (PBM, PDBM, BMF) was marked by presence of fatty acids, ketones and some terpenoids. The profile of human milk-based products (RTF and HMF) was more similar to pasteurized breastmilk than to preterm breastmilk, but presented more aromatic hydrocarbons, terpenoids and specific fatty acids, whereas the profile of infant formulas was characterized by alkyls, aldehydes and furans.

Breastmilk is considered a bridge between the antenatal and postnatal chemosensory environment. Maternal diet and metabolism are known to influence compounds in amniotic fluid and breastmilk, suggesting that some substances might be common to both (19, 22, 33). The many chemical reactions that occur in foodstuffs involving metabolites, including proteins, sugars and lipids, contribute to the profile of volatile compounds and can potentially lead to formation of either pleasant or off-flavors in the food matrix (4, 34, 35). Previous studies reported that the volatile profile of breastmilk is more variable than of infant formulas due to individual contributions from endogenous metabolism and diet (24, 25, 36). On one hand, the profile of volatiles in infant formulas is reported to be characterized by compounds developed during the manufacturing process (involving heat, light and storage) generating alkanes, aldehydes and furans and present less terpenoids (24, 26). On the other hand, it has been suggested that volatile compounds in breastmilk result from oxidation of lipids (yielding ketones, aldehydes, free fatty acids, and fatty acid esters) and maternal diet (mostly terpenoids) (24, 25, 36). Thus, our findings are largely in agreement with previous reports. Furthermore, we report for the first time the volatile profile of fortified breastmilk and of products made from pasteurized donor breastmilk, adding new information to human milk research.

The varying distribution of volatile compounds in milk might provide different cues related to feeding behavior. It has been demonstrated that exposure to aroma of breastmilk and infant formula evokes different physiological reactions in infants (6, 7). In a small study, Bingham et al. (7) investigated the impact of olfactory exposure during tube-feeding on non-nutritive sucking and reported increased sucking behavior (measured by number of sucks and suck bursts) in preterm infants exposed to smell of fortified breastmilk compared to infants exposed to smell of infant formula (7). Aoyama et al. (6) reported a significant increase in oxygenation of the olfactory region of the brain following exposure to smell of breastmilk but not to formula. Preterm infants also have functional olfactory receptors (14) and there is an emerging interest in potential health benefits related to sensory exposure to smell and taste of milk in tube fed preterm infants (12). However, most studies investigating physiological and behavioral changes in response to smell of milk have not simultaneously assessed the volatile compounds that could be responsible for the effects seen, which prevents us from inferring which compounds detected in our study may have the potential to influence sucking behavior or changes in cerebral oxygenation.

The volatile profile observed in fortified breastmilk retrieved from a hospital refrigerator is particularly important for nutrition in preterm infants. To ensure a continuous human milk-based diet with sufficient nutrition for their preterm infants, mothers often need to express their milk, fortify and refrigerate it (30). Even though changes in the chemical properties of breastmilk following fortification have been reported previously (37–39), fortification of breastmilk is extremely important for nutrition and growth of some preterm infants (40). Compared to preterm breastmilk, our data demonstrate that the volatile profile of breastmilk with bovine milk-based fortifier (BMF) is marked by products generated through lipolysis (fatty acids) and lipid auto-oxidation (ketones and aldehydes), suggesting that changes in breastmilk properties occur within the recommended refrigerated storage period for fortified breastmilk (30). Additionally, volatile compounds in breastmilk with human milk-based fortifier breastmilk (HMF) differ from BMF and preterm breastmilk, with higher concentrations of aromatic hydrocarbons, terpenes, alkyls, alcohols, and some fatty acids. Although no harm has been directly associated with fortification of breastmilk, some studies have suggested a relationship between bovine milk-based diet and milk-curd obstruction (41, 42) and necrotising enterocolitis (43, 44) in very preterm infants. However, the underlying mechanism leading to formation of curds in fortified breastmilk is not completely understood.

The majority of the volatile compounds detected in our study are oxidation products of the polyunsaturated fatty acid (PUFA) moieties of lipids. Hexanal and pentanal are products of the oxidation of linoleic acid and are considered markers of lipid oxidation in human milk and infant formula (27, 35). Nonanal is a product of the oxidation of *n*-9 fatty acids such as oleic acid, the most abundant fatty acid in breastmilk (35). Relative abundance of hexanal, pentanal, octanal, and benzaldehyde were significant higher in both powder and liquid formula, whereas nonanal, 3-methyl butanal, and 2-hexenal were significant higher in breastmilk with bovine-milk based fortifier. Propanal, pentanal, and hexanal previously have been linked to degradation of powdered infant formula, mostly due to thermal processing and storage conditions (i.e., light, temperature and length of storage) (26, 27).

Increasing concentrations of aldehydes and free fatty acids following storage of breastmilk at 4°C and −19°C have been reported previously (4, 45), and a rise in concentration of free fatty acids (FFA) due to enzymatic lipolysis might be responsible for conferring an unpleasant odor to breastmilk stored under refrigerated conditions (31, 45). In fact, Spitzer et al. reported a 5-fold increase in the concentration of octanoic acid after 1 day of refrigerated storage (45). Compared to pasteurized donor breastmilk and preterm breastmilk that had been stored frozen at −80°C, samples of BMF refrigerated for at least 8 h presented a much higher concentration of all FA (including octanoic acid) and the majority of fatty acid esters and aldehydes detected in our study. One possible explanation is that FA and FA esters result from the activity of breastmilk lipase during refrigerated storage of untreated breastmilk, which does not occur in pasteurized breastmilk due to inactivation of lipase during the pasteurization process (46). Additionally, it is possible that the lower concentration of aldehydes in preterm breastmilk relates to the fact that samples were frozen immediately after collection. The impact of different fortification and storage practices for fortified breastmilk on feed tolerance and infant nutrition needs further investigation.

To the best of our knowledge, few studies previously have compared the volatile profile of liquid and powdered formulas (24, 47). In general, previous studies indicate that the volatile profile of liquid formulas is rich in terpenoids and ketones, whereas powder formulas are characterized by the presence of aldehydes resulting from lipid oxidation and thermal degradation generated during manufacturing and storage (24, 47), which might confer an unpleasant odor to milk (27, 35). The extreme heat used for water evaporation during powder formula production might intensify lipid auto-oxidation and contribute to formation of aldehydes (24). Our results substantiate this, showing higher abundance of 2-butanone, acetone, benzaldehyde, 2-ethyl-furan, and pentane in liquid formula, whereas aldehydes such as hexanal, pentanal, and octanal contributed significantly to the volatile profile of powder formulas. Except for terpenoids, which in our study were more abundant in powder compared to liquid formula, the differences observed between the two formula types are in line with previous reports (24, 47) and probably relate to different farming practices, formulation, manufacturing, storage, and packaging processes in production of powdered and liquid infant formulas. Interestingly, and contrary to what might be expected, powder formulas made from different protein sources (soy, goat, and bovine milk) exhibit similar volatile profiles.

The presence of terpenoids in breastmilk and formula has been reported previously (24, 47) and likely originates from maternal diet, skin absorption of cosmetics or inhalation of products containing terpenoid-based fragrancies (48, 49), animal diet/pasture (32, 50), and from mixed vegetable oils added to infant formulas to enrich them with essential fatty acids and liposoluble vitamins. We detected eight different terpenoids, largely in breastmilk samples and especially among the human milk-based products (HMF and RTF) in which, compared to other milk types, significantly higher relative peak areas were observed for half of the terpenoids. In quantifying volatile compounds in breastmilk, liquid and powder formulas, Hausner et al. (24) concluded that the main source of terpenoids in breastmilk is direct transfer from maternal diet, with high individual variability. It seems likely that, because human milk-based products are made from pooled donor breastmilk, there could be contributions from many different donors to the overall terpenoid content in these products.

Ketones are also generated from lipid oxidation, Maillard Reactions (non-enzymatic browning of carbohydrates) and thermal processing (34). In our study, ketones were detected in almost all milk types analyzed, but acetone, pinacolone and 2-butanone were more abundant in infant formulas than in breastmilk samples, consistent with previous findings (25, 26, 47). These compounds are particularly odor active and are thought to contribute to the overall aroma of human milk and infant formulas (26, 51). Similarly, furans are formed by Maillard Reactions, thermal degradation of some amino acids, oxidation of PUFAs, carotenoids and ascorbic acid (52, 53), from spray-drying and hydrolysis of milk for infant formula production (52). 2-ethylfuran and 2-pentylfuran are known products of lipid oxidation and previously have been detected in both breastmilk and infant formula (24). Furans have been described as “possibly carcinogenic to humans” and their presence in food is monitored globally (54, 55). Some studies reported concentrations in infant formulas ranging from 0.02 to 36 μg.g^−1^ (52, 56), but estimated margin of exposure through consumption of infant formula was not considered of high concern (57). In our study furans were detected in most milk types but 2-ethyl-furan and 2-butyltetrahydro-furan were significantly more abundant in infant formulas.

Significant higher relative peak areas of the aromatic hydrocarbons styrene, indane, *ortho* and *meta* cymene, three isomers of ethyl methyl benzene and 1,3-bis(1,1-dimethylethyl)-benzene were observed in human milk-based products. These compounds have been previously found in samples of breastmilk and are likely to originate from exposure to pyrogenic and petrogenic air pollutants (58–60). Volatile organic compounds (VOCs) have been highly correlated with in-door air exposure to VOCs, and inhalation is thought to be the main entry route into the human body (58–60). However, the levels of VOCs reported were not deemed to be of health concern. As for terpenoids, a possible reason for the observed concentrations of specific VOCs in the human milk based products (HMF and RTF) could be the contribution of multiple donors living in areas of high pollution.

Liquid and powder formulas presented the highest relative abundance of aldehydes, consistent with previous reports (26, 27), but also the lowest relative abundance of fatty acid esters and fatty acids. Aldehydes generally present lower odor thresholds compared to fatty acids (45). Even low concentrations of compounds with a low odor threshold may be sufficient to provoke odor detection and confer off-notes to food (27), indicating that it is possible that the smell of infant formula is less pleasant to babies compared to the smell of fresh breastmilk. The odor activity values of several aldehydes found in fresh, refrigerated and frozen breastmilk have been reported to remain constant in breastmilk stored under refrigerated conditions (45) but to increase in breastmilk frozen for 6 months (4). In contrast, the OAV of most fatty acids increased with refrigeration/frozen storage (4, 45). Most OAV in fresh breastmilk reported in these studies were <1 (4, 45), suggesting that smell of fresh breastmilk is not very intense. Nevertheless, it is worth mentioning that only an approximated OAV for breastmilk was reported, calculated based on odor threshold information from adults (4, 45), which might differ from that in babies. Further, odor threshold and odor release are highly influenced by the composition of the matrix under investigation (skim milk, whole milk, water, air) (61). Thus, odor thresholds and OAV of volatile compounds in infant formula may differ from those in human milk, and remain to be determined.

The concentration and interactions of odor-active compounds is very relevant to the overall aroma of food, to palatability (34), and can even influence infants' acceptance of weaning foods (62). From the compounds identified in our study, it seems likely that aldehydes and ketones are responsible for most of the olfactory cues in infant formulas whereas FA and FA esters contribute most to the smell of breastmilk. The aldehydes and ketones have a relatively low odor threshold, meaning that they can be perceived even at low concentrations, and are responsible for malty, almond, fatty, and even fishy odor descriptions (27, 45). Most fatty acids are important odor-active compounds but have high odor perception thresholds (at parts per million level) (63). In contrast, esters of fatty acids present lower perception threshold (at parts per billion level) but their contribution to overall flavor perception can be potentiated by concentration and interaction with other esters and volatile compounds (64). For example, at low concentration, esters can contribute positively to aroma conferring fruit-like notes, but at high concentrations they may introduce an unpleasant flavor impression (32). Thus, not only odor activity of a specific compound but also the interaction with other volatile compounds in the food matrix, play a role in overall olfactory perception.

Infants may have a greater sensitive olfactory perception than adults, which would allow detection of odors even at low concentrations (4). Given that olfactory perception occurs retronasally in the oropharyngeal cavity, as well as nasally, the feeding experience *per se* might intensify the sensory perception of odorants in milk, even at low odor concentrations (65). Our findings demonstrate that the compounds responsible for the smell of preterm breastmilk differ from those of other options commonly used for feeding preterm infants. Whether these differences have any biological or physiological effects upon preterm infant nutrition requires further investigation. Sensory stimulation with smell and taste of milk during tube feeds is a very simple intervention and might be an important factor in stimulation of the cephalic phase response, assisting with tolerance to tube feeds. The results from two ongoing randomized clinical trials will contribute to better understanding of the impact of exposure to smell and taste on nutrition of preterm infants (66, 67).

### Strengths and Limitations

Some limitations to this study should be mentioned. First, we were unable to analyze samples of preterm breastmilk in duplicate due to limited volume. However, triplicate measurements on other milk types were highly reproducible (data not shown) and suggest this limitation was minor. Secondly, samples of fresh fortified breastmilk were not obtained given the importance of even small amounts of this milk for preterm infants in the Neonatal Intensive Care Unit. Ethical approval was obtained only to retrieve samples that exceeded the unit's policy for refrigerated storage of fortified breastmilk and were therefore no longer clinically useful. Thus, despite differences observed between the profile of volatile compounds of BMF and unfortified preterm breastmilk, we are unable to comment on whether freshly fortified breastmilk contains different volatile components from fortified breastmilk that has been refrigerated for 8 h. Additionally, no maternal nutritional information was collected, meaning we cannot relate maternal diet to the volatile profile found in breastmilk samples. Finally, we opted for an untargeted approach for the detection of volatile compounds; thus, quantification of specific compounds was only relative and could have been influenced by the matrix effects between the internal standard concentration and fat content of samples, as previously reported by Elisia and Kitts (35). We did not analyze macronutrient composition of breastmilk samples due to limited volume available and the incompatibility of human milk analyzers with fortified breastmilk and infant formulas (68). Nevertheless, the volatile profile of a biological sample results from complex physical, chemical, and biological interactions. An untargeted approach is therefore essential to investigate the contribution of diverse sensory active compounds to the organoleptic properties of matrices such as human milk (26), and may serve as screening step for compounds that require further investigation and absolute quantification.

Previous research into odorants of human milk has been carried out using techniques that require use of solvents (4, 31, 45), distillation (25) or extraction of volatile compounds applying high temperatures (69) to increase volatility of compounds, all of which may alter the chemical properties of milks and, therefore, analyze odorants that do not reflect exactly what infants are exposed to through feeding. In contrast, head space SPME-GC-MS does not use solvent and allows the identification of volatile compounds without requiring the SPME fiber to come into contact with the actual sample matrix, but only with the volatile compounds trapped in the headspace above the sample (26, 58). Thus, this approach that extracts volatile compounds in milk at temperatures similar to feeding temperature might provide a better understanding of sensory active compounds that could play a physiological role in sensory stimulation in the newborn. Moreover, for the first time the volatile compounds in human milk-based products and fortified breastmilk were analyzed.

### Implications/Future Directions

Given that provision of smell and taste of milk with tube feeds is a very simple and non-invasive intervention that might enhance metabolism of feeds, future research is needed to understand whether sensory active volatile compounds in milk can activate the cephalic phase response of digestion and hence improve feed tolerance in preterm infants. Furthermore, we have observed products from lipid oxidation and lipase activity in samples of breastmilk with bovine milk-based fortifier that had been refrigerated for at least 8 h, suggesting that changes in breastmilk properties can occur during the recommended storage of fortified breastmilk. Thus, how fortification and storage practices impact on digestion of fortified breastmilk merits further investigation. The detection of potential environmental contaminants such as aromatic hydrocarbons and furans in almost all milk types analyzed is potentially of concern, particularly as these were highest in human milk-based products made from pooled donor breastmilk. The level of potential exposure of preterm infants receiving these products needs to be determined so that any risk can be assessed.

## Conclusions

Sensory active products of fatty acids oxidation are the major contributors to olfactory cues in infant feeds. We have demonstrated that the profile of volatile compounds in preterm breastmilk differs from of other milks commonly used for infant feeding but the physiological impact of these differences in the nutrition of preterm infants requires further research. In addition, analysis of volatile compounds may be useful for monitoring oxidation in milk and detection of environmental contaminants.

## Data Availability Statement

The raw data supporting the conclusions of this article will be made available by the authors, without undue reservation.

## Ethics Statement

The studies involving human participants were reviewed and approved by New Zealand's Health and Disability Ethics Committee. The patients/participants provided their written informed consent to participate in this study.

## Author Contributions

MM designed the research question, assisted with method development, carried out laboratory and statistical analysis, interpreted the results, and drafted the manuscript. CP assisted with method development, laboratory and statistical analysis, contributed to interpretation of results and the manuscript development. FB helped design the research question, contributed to interpretation of results and the manuscript development. JH helped design the research question, contributed to interpretation of results and edited the final manuscript. SP assisted with method development and edited the final manuscript. All authors contributed to the article and approved the submitted version.

## Conflict of Interest

Human milk-based products were donated by Prolacta Bioscience but the company had no influence in the analysis and interpretation of the results presented here. SP is currently employed by Fonterra but was a researcher at the Liggins Institute during her involvement in this research. The remaining authors declare that the research was conducted in the absence of any commercial or financial relationships that could be construed as a potential conflict of interest.
