# Supplementary material for: Olfactory Cues in Infant Feeds: Volatile Profiles of Different Milks Fed to Preterm Infants
Source: Front Nutr. 2021 Jan 15;7:603090. doi: 10.3389/fnut.2020.603090 (PMC7843498; doi:10.3389/fnut.2020.603090)
Supplement: Supplementary file 1 [file Table_1.docx]

**Supplementary Table 1** - Relative peak area of volatile compounds in different milk types.

| Compounds | RT  (min) | Ref Ion  (m/z) | PBM  (n=15) | PDBM  (n=4) | BMF  (n=10) | HMF  (n=2) | RTF  (n=3) | LF  (n=6) | PF  (n=7) | FDR  *p*-value |
| --- | --- | --- | --- | --- | --- | --- | --- | --- | --- | --- |
| **Alcohol** |  |  |  |  |  |  |  |  |  |  |
| ***methanol**** | 1.82 | 31 | 24621  ±43118 | 20167  ±37469 | 7474  ±13854 | - | - | 1667  ±3727 | - | ns |
| ethanol | 1.94 | 45 | 8367 ^a c^  ±17050 | 527 ^a c^  ±823 | 38791 ^b^  ±38316 | 33391 ^a b^  ±25381 | 42797 ^a b c^  ±36554 | 799 ^a c^  ±1123 | 306 ^a c^  ±344 | <0.001^A^ |
| ***isopropanol**** | 2.07 | 45 | - | - | 128 ^a^  ±406 | 93802 ^b^ ±123861 | 45369 ^b^  ±28743 | 728 ^a^  ±1628 | - | <0.001^B^ |
| 2-methylpropanol | 2.15 | 59 | 31951  ±69016 | 295  ±344 | 3846  ±8156 | 89  ±126 | 69  ±77 | 637  ±757 | 5  ±6 | ns |
| 1-octenol | 12.98 | 57 | 320 ^a^  ±572 | 1902 ^a b^  ±1785 | 1088 ^a b^  ±1751 | 2065 ^a b^  ±86 | 1006 ^a b^  ±650 | 265 ^a b^  ±314 | 3959 ^b^  ±3849 | <0.001^B^ |
| 2-ethylhexanol | 14.51 | 57 | 1026 ^a b d^  ±3121 | 54 ^a b d^  ±100 | 57 ^a b d^  ±94 | 5192 ^c d^  ±2534 | 16331 ^c^  ±14367 | 26692 ^b c^  ±36298 | 28 ^b d^  ±31 | <0.001^B^ |
| **Aldehyde** |  |  |  |  |  |  |  |  |  |  |
| ***3-methyl butanal**** | 3.11 | 43 | - | 41 ^a^  ±83 | 2473 ^b^  ±2107 | - | - | 763 ^a c^  ±1528 | 609 ^b c^  ±685 | <0.001^B^ |
| ***pentanal**** | 3.9 | 44 | 6861 ^a c^  ±10498 | 3543 ^a c^  ±3512 | 31973 ^b c^  ±20282 | 12103 ^a b c^  ±346 | 5326 ^a^  ±4601 | 11325 ^a c^  ±15754 | 380083 ^b c^  ±378430 | <0.01^B^ |
| 2-pentenal | 5.98 | 83 | 101 ^a^  ±232 | 81 ^a^  ±162 | 469 ^a^  ±866 | 169 ^a b^  ±6 | 113 ^a b^  ±100 | 591 ^a b^  ±726 | 3060 ^b^  ±3156 | <0.001^A^ |
| ***hexanal**** | 6.56 | 56 | 65621 ^a^  ±158777 | 100447 ^a b^  ±57284 | 105252 ^a b^  ±100365 | 60598 ^a b^  ±10180 | 55370 ^a b^  ±26068 | 17589 ^a^  ±27835 | 688518 ^b^  ±675367 | <0.001^A^ |
| ***2-hexenal**** | 9.05 | 55 | 41^a^  (96) | 44 ^a b^  (88) | 731 ^b^  (887) | 237 ^a b^  (6) | 227 ^a b^  (92) | - | 485 ^b^  (344) | <0.001^A^ |
| ***heptanal**** | 9.82 | 43 | 799  ±1582 | 3038  ±1599 | 6561  ±8423 | 4932  ±814 | 2599  ±396 | 936  ±1851 | 4686  ±3235 | ns |
| (Z)-2-heptenal | 12.38 | 83 | 128  ±229 | 322  ±304 | 1123  ±1923 | 582  ±5 | 317  ±355 | - | 4542  ±5063 | ns |
| benzaldehyde | 12.70 | 106 | 73 ^a^  ±150 | 233 ^a b^  ±150 | 1098 ^b^  ±1351 | - | 416 ^a b^  ±398 | 9016 ^c^  ±9109 | 3266 ^b c^  ±3350 | <0.001^A^ |
| octanal | 13.05 | 56 | 247 ^a^  ±453 | 1005 ^a b^  ±1317 | 1231 ^b^  ±808 | 535 ^a b^  ±92 | 597 ^a b^  ±209 | 181 ^a b^  ±292 | 4375 ^c^  ±4107 | <0.001^A^ |
| (E,E)-2,4-heptadienal | 14.1 | 81 | 87  ±181 | - | 102  ±191 | - | - | - | 2372  ±2661 | ns |
| (E)-2-octenal | 15.45 | 83 | 102 ^a^  ±185 | 5 ^a^  ±10 | 590 ^a^  ±752 | - | 103 ^a^  ±90 | - | 982 ^b^  ±1082 | <0.001^A^ |
| nonanal | 16.08 | 82 | 137 ^a^  ±270 | 136 ^a^  ±200 | 9077 ^b^  ±11972 | 421 ^a^  ±114 | 1145 ^a b^  ±858 | 292 ^a^  ±652 | 545 ^a^  ±601 | <0.001^A^ |
| **Alkyls** |  |  |  |  |  |  |  |  |  |  |
| pentane | 1.8 | 43 | 6078  ±7156 | 4228  ±3735 | 6033  ±8346 | 3722  ±1560 | 829  ±740 | 65510  ±68377 | 62545  ±66402 | ns |
| ***undecane**** | 13.34 | 57 | 256  ±462 | 374  ±713 | 745  ±1186 | - | 374  ±648 | 150  ±336 | 198  ±223 | ns |
| ***tridecane**** | 19.07 | 73 | - | - | - | - | 68±69 | - | - | <0.001^B^ |
| ***nonane**** | 7.09 | 57 | - | 48  ±57 | 434  ±734 | - | - | 129  ±199 | 111  ±125 | ns |
| 2-methyl pentane | 1.92 | 71 | 10005  ±24710 | 651  ±782 | 2038  ±4291 | - | - | 31238  ±47953 | 564  ±634 | ns |
| cyclohexane | 2.05 | 56 | 7667  ±17632 | 3203  ±5585 | 346  ±792 | - | - | 1721  ±2893 | 1408  ±1582 | ns |
| hexyl-cyclohexane | 17.54 | 83 | - | - | - | 129 ^a^  ±68 | 612 ^a^  ±326 | - | 45 ^a^  ±51 | <0.001^B^ |
| butyl-cyclohexane | 11.44 | 83 | - | - | - | 208 ^a^  ±71 | 1065 ^a^  ±160 | - | - | <0.001^A^ |
| 1,2,4-trimethyl-cyclopentane | 4.27 | 70 | 1268 ^a^  ±3287 | 9590 ^b^  ±5824 | 1070 ^a^  ±1123 | 3561 ^a^  ±366 | 1383 ^a^  ±1181 | 1865 ^a^  ±1356 | 3272 ^a^  ±3531 | <0.001^A^ |
| 5-methyl-1-heptene | 4.31 | 55 | 1494  ±3256 | 2890  ±1983 | 1540  ±1012 | 3911  ±2170 | 2651  ±2614 | 1873  ±2241 | 2011  ±2260 | ns |
| 2,4-dimethyl-1-heptene | 5.6 | 43 | 5158  ±7923 | 1035  ±714 | 2452  ±5916 | 664  ±251 | - | 17609  ±27444 | 8053  ±8957 | ns |
| 2,3-dimethyl octane | 9.02 | 57 | 9  ±36 | 46  ±59 | 269  ±520 | 297  ±261 | 2075  ±1198 | 219  ±269 | 7525  ±8381 | ns |
| 2-methyl nonane | 9.1 | 57 | - | 366  ±342 | 812  ±1590 | 162  ±53 | 54  ±94 | 892  ±1318 | 288  ±323 | ns |
| 2,3-dimethyl-butane | 1.916 | 43 | 18505  ±45450 | 957  ±1134 | 4869  ±7736 | 2712  ±2762 | 2289  ±3294 | 19149  ±25558 | 4023  ±4526 | ns |
| 2,3,5-trimethyl-hexane | 4.78 | 43 | 4582  ±7300 | 2839  ±2334 | 25516  ±50745 | 585  ±347 | 64  ±110 | 3949  ±3599 | 9491  ±10507 | ns |
| 2,3-dimethyl-heptane | 6 | 85 | 953  ±1571 | 1080  ±923 | 1549  ±2969 | 631  ±47 | 29  ±50 | 6405  ±6973 | 454  ±511 | ns |
| 3-ethyl-2-methyl-1,3-hexadiene | 14.23 | 67 | 126 ^a^  ±271 | 463 ^a b^  ±208 | 509 ^a b^  ±995 | 519 ^a b^  ±28 | 358 ^a b^  ±175 | 79 ^a b^  ±144 | 2152 ^b^  ±2089 | <0.01^A^ |
| branched C9 alkane | 7.00 | 113 | - | - | - | - | - | - | 345  ±388 | ns |
| branched C10 alkane A | 9.35 | 57 | 60  ±232 | 28  ±56 | 307  ±278 | 657  ±68 | 933  ±638 | 2289  ±4239 | 1522  ±1712 | ns |
| branched C10 alkane B | 9.85 | 56 | 84  ±243 | 1780  ±1194 | 1668  ±2398 | 2160  ±204 | 802 ±  452 | 3842  ±7455 | 3393  ±3611 | ns |
| branched C10 alkane | 10.00 | 57 | 44  ±135 | 48  ±86 | 84  ±110 | - | 173  ±291 | - | 315  ±354 | ns |
| branched C11 alkane A | 10.91 | 57 | 10  ±40 | 461  ±481 | 414  ±781 | 290 ±  57 | 138  ±119 | 779  ±926 | 337  ±380 | ns |
| branched C12 alkane | 18.40 | 55 | - | 11  ±22 | 225  ±380 | - | 43  ±47 | - | 235  ±265 | ns |
| **Aromatic Hydrocarbon** |  |  |  |  |  |  |  |  |  |  |
| toluene | 4.69 | 91 | 1462  ±5562 | 116  ±231 | 1674  ±4636 | - | - | 1740  ±3748 | 3372  ±3793 | ns |
| m-xylene | 7.32 | 91 | 113  ±122 | 28939  ±56445 | 1785  ±2621 | 2361  ±78 | 1829  ±824 | 2704  ±5517 | 114  ±128 | ns |
| o-xylene | 7.57 | 91 | 258  ±892 | 13592  ±25919 | 200  ±169 | 2073  ±1627 | 3974  ±1715 | 1852  ±3128 | 175  ±197 | ns |
| p-xylene | 8.46 | 106 | 91  ±351 | 313  ±81 | 94  ±219 | 653  ±335 | 884  ±652 | 542  ±869 | 46  ±52 | ns |
| o-cymene | 13.58 | 119 | - | - | - | 462 ^a^  ±24 | 657 ^a^  ±474 | - | 20 ^b^  ±23 | <0.001^B^ |
| m-cymene | 14.48 | 119 | - | - | - | 237 ^a^  ±102 | 597 ^a^  ±589 | - | 43 ^b^  ±48 | <0.001^A^ |
| styrene | 8.76 | 104 | 228 ^a^  ±768 | 3223 ^b^  ±1034 | 1620 ^a b^  ±3039 | 4101 ^b^  ±2310 | 1992 ^a b^ ±1432 | - | - | <0.001^A^ |
| ethyl methyl benzene A | 10.65 | 105 | 28 ^a^  ±80 | 98 ^a^  ±133 | 49 ^a^  ±93 | 5021 ^b^  ±2529 | 6053 ^b^  ±7494 | 53 ^a^  ±119 | 35 ^a^  ±39 | <0.001^A^ |
| ethyl methyl benzene B | 11.25 | 105 | 37 ^a^  ±144 | 5 ^a^  ±10 | - | 790 ^b^  ±307 | 1567 ^b^  ±1340 | - | - | <0.001^A^ |
| ethyl methyl benzene C | 11.64 | 105 | 71 ^a^  ±219 | 107 ^a^  ±69 | 47 ^a^  ±74 | 7990 ^b^  ±2398 | 12611 ^b^  ±10222 | 89 ^a^  ±133 | 43 ^a^  ±48 | <0.001^A^ |
| indane | 13.12 | 117 | - | - | - | 409 ^a^  ±28 | 559 ^a^  ±335 | - | - | <0.001^B^ |
| 1,3-bis(1,1-dimethylethyl)-benzene | 18.88 | 175 | - | 167 ^a^  ±256 | 673 ^a^  ±767 | 401 ^a^  ±109 | 347 ^a^  ±198 | - | 15 ^b^  ±17 | <0.001^B^ |
| **Fatty acids** |  |  |  |  |  |  |  |  |  |  |
| ***butanoic acid**** | 9.9 | 60 | 378 ^a^  ±559 | 268 ^a^  ±352 | 3953 ^a b^  ±6269 | 1431 ^b^  ±804 | 1014 ^a b^  ±870 | - | - | <0.01^B^ |
| ***hexanoic acid**** | 15.54 | 60 | 1406  ±4071 | 797  ±1587 | 2816  ±3827 | 2548  ±105 | 4509  ±3101 | - | - | ns |
| ***octanoic acid**** | 20.71 | 60 | 2887  ±9442 | - | 792601  ±1495737 | 219  ±309 | 2337  ±1111 | 2564  ±5636 | - | ns |
| nonanoic acid | 23.13 | 129 | - | - | 268  ±490 | - | - | - | - | ns |
| n-decanoic acid | 25.43 | 73 | - | - | 4445  ±9722 | - | - | - | - | ns |
| **Fatty acid esters** |  |  |  |  |  |  |  |  |  |  |
| branched C8 fatty acid ester | 18.06 | 88 | - | 236 ^a^  ±424 | 30973 ^b^  ±49605 | 625 ^b^  ±98 | 1087 ^b^  ±678 | 108 ^a^  ±243 | - | <0.001^B^ |
| dimethyl carbonate | 2.72 | 59 | 284  ±972 | 24  ±42 | 121  ±267 | 71  ±101 | 60  ±75 | 122  ±185 | 1  ±1 | ns |
| ***methyl butyrate**** | 4.04 | 74 | 292 ^a^  ±599 | 309 ^a^  ±152 | 1167 ^b^  ±799 | 354 ^a b^  ±148 | 136 ^a b^  ±120 | 35 ^a^  ±79 | 62 ^a^  ±69 | <0.001^A^ |
| ***ethyl butyrate**** | 6.03 | 88 | - | 176  ±353 | 1127  ±3061 | 473  ±162 | 552  ±483 | - | - | ns |
| ***isopropyl butyrate**** | 7.15 | 89 | - | - | - | 391 ^a^  ±172 | 807 ^a^  ±717 | - | - | <0.001^B^ |
| vinyl caproate | 12.81 | 43 | 931  ±1836 | 792  ±528 | 2030  ±2466 | 486  ±35 | 480  ±358 | - | 6974  ±7761 | ns |
| ***methyl octanoate**** | 16.1 | 115 | 980  ±2158 | 178  ±347 | 60508  ±108415 | - | - | 173  ±386 | 31  ±35 | ns |
| 2-ethylhexyl acetate | 16.86 | 70 | - | - | 70  ±101 | 360  ±153 | 1196  ±1447 | 270  ±605 | 56  ±62 | ns |
| ***methyl decanoate**** | 21.48 | 143 | 276 ^a^  ±524 | - | 6473 ^b^  ±8922 | - | - | 52 ^a^  ±117 | - | <0.001^B^ |
| ethyl decanoate | 23.23 | 88 | - | 18 ^a^  ±36 | 633 ^b^  ±808 | 240 ^b c^  ±128 | 166 ^c^  ±15 | - | - | <0.001^B^ |
| ***methyl dodecanoate**** | 26.34 | 74 | 106 ^a^  ±202 | - | 277 ^b^  ±265 | - | - | - | - | <0.001^B^ |
| ***methyl hexanoate**** | 9.99 | 74 | 698 ^a c^  ±1997 | 3782 ^a b^  ±5896 | 4442 ^b c^  ±4483 | 1746 ^a b^  ±104 | 392 ^a b c^  ±347 | 110 ^a c^  ±168 | 87 ^a c^  ±98 | <0.001^A^ |
| isopropyl hexanoate | 13.34 | 99 | - | - | - | 469 ^a^  ±308 | 675 ^a^  ±575 | - | - | <0.001^B^ |
| **Furans** |  |  |  |  |  |  |  |  |  |  |
| 2-ethyl-furan | 3.21 | 81 | 144 ^a^  ±237 | 214 ^a b^  ±202 | 168 ^a b^  ±202 | 1047 ^a b^  ±1047 | 429 ^a b^  ±306 | 4902 ^b^  ±7041 | 3044 ^b^  ±4662 | <0.001^A^ |
| 2-pentyl-furan | 11.32 | 81 | 169  ±377 | 830  ±451 | 536  ±308 | 2431  ±354 | 2348  ±1368 | 3493  ±2772 | 4934  ±1116 | ns |
| 2-propyl-furan | 14.59 | 81 | 36  ±138 | - | 63  ±93 | 72  ±59 | 267  ±123 | 317  ±495 | 2711  ±4806 | ns |
| 2-butyltetrahydro-furan | 16.82 | 71 | 35 ^a^  ±81 | - | 98 ^a b^  ±106 | 143 ^a b^  ±53 | 437 ^b^  ±341 | 5 ^a^  ±10 | 1360 ^b^  ±1879 | <0.001^A^ |
| **Ketones** |  |  |  |  |  |  |  |  |  |  |
| ***acetone**** | 2.04 | 43 | 66133  ±99397 | 11019  ±8947 | 76902  ±100357 | - | - | 492890  ±774822 | 51702  ±51617 | ns |
| 2-butanone | 2.55 | 43.1 | 9503 ^a^  ±20443 | 3278 ^a c d^  ±455 | 44831 ^c^  ±52922 | 2180 ^a^  ±337 | 1909 ^a^  ±705 | 932182 ^b^  ±735935 | 55056 ^c d^  ±61772 | <0.001^B^ |
| 2-(1,1-dimethylethyl)-cyclobutanone | 3.01 | 83 | 3777  ±12147 | 318  ±180 | 338  ±825 | 125  ±9 | 163  ±129 | 2075  ±2114 | 964  ±994 | ns |
| 1-penten-3-one | 3.80 | 55 | 5873  ±12823 | 9  ±17 | 5370  ±14180 | 584  ±24 | 394  ±621 | - | 4998  ±4653 | ns |
| 3,3-dimethyl-2-butanone (pinacolone) | 4.18 | 57 | 745 ^a^  ±1765 | 1090 ^a^  ±1271 | 1134 ^a^  ±1155 | 3230 ^a b^  ±1308 | 1199 ^a b^  ±404 | 97 ^a^  ±138 | 23814 ^b^  ±25072 | <0.001^A^ |
| 2-heptanone | 9.72 | 58 | 93  ±187 | 1313  ±664 | 10139  ±24397 | 992  ±384 | 931  ±298 | 11396  ±10251 | 956  ±1066 | ns |
| 6-methyl-2-heptanone | 11.78 | 58 | 60  ±149 | 344  ±101 | 242  ±462 | 325  ±69 | 82  ±71 | 171  ±237 | - | ns |
| 1-octen-3-one | 12.47 | 97 | 134  ±316 | 62  ±65 | 242  ±255 | 114  ±5 | 131  ±25 | 11  ±25 | 294  ±282 | ns |
| 3-octanone | 12.6 | 43 | 152  ±320 | 182  ±204 | 359  ±470 | 60  ±84 | 9  ±16 | - | 58  ±65 | ns |
| 2-nonanone | 15.94 | 127 | - | - | 9 ±24 | - | - | - | - | ns |
| 3,5-octadien-2-one | 16.1 | 95 | 9  ±35 | 63  ±126 | 116  ±246 | 692  ±5 | 515  ±309 | - | 2055  ±2312 | ns |
| acetophenone | 16.18 | 105 | 8 ±20 | 28 ±33 | 155 ±236 | 147 ±45 | 261 ±208 | 219 ±165 | 87 ±98 | ns |
| **Siloxane** |  |  |  |  |  |  |  |  |  |  |
| hexamethyl disiloxane | 2.5 | 147 | 380  ±772 | - | 35  ±111 | 578  ±176 | 269  ±239 | - | - | ns |
| trimethyl-silanol | 2.67 | 75 | 20605  ±58620 | 3943  ±4160 | 6533  ±8896 | 6404  ±6917 | 4390  ±2574 | - | 8  ±9 | ns |
| 1,1,3,3,5,5-hexamethyl- trisiloxane | 3.98 | 193 | - | 393 ^a^  ±786 | - | - | 801 ^b^  ±1247 | - | - | <0.001^B^ |
| hexamethyl- cyclotrisiloxane | 5.03 | 207 | 16641 ^a^  ±25189 | 93652 ^a^  ±107786 | 8477 ^a^  ±20017 | 7514 ^a^  ±5465 | 12090 ^a^  ±8777 | - | - | <0.05^B^ |
| tetramethyl- silane | 8.3 | 77 | 44475  ±111006 | - | 1365  ±4317 | - | 17637  ±25004 | - | 34252  ±38533 | ns |
| dimethyl- silanediol | 8.6 | 77 | 3727  ±11994 | - | 1676  ±5300 | - | 24548  ±42519 | - | 14203  ±15978 | ns |
| siloxane A | 9.26 | 267 | 62 ^a^  ±123 | 423 ^a^  ±581 | 140 ^a^  ±303 | 6069 ^b^  ±2545 | 6400 ^b^  ±3449 | - | - | <0.001^A^ |
| octamethyl-cyclotetrasiloxane | 10.27 | 281 | 5102  ±11670 | 32051  ±63756 | 711  ±1724 | 31987  ±10800 | 58645  ±43021 | - | - | ns |
| siloxane B | 13.61 | 193 | 57  ±115 | 5  ±9 | 37  ±74 | - | 15  ±16 | 31  ±66 | 115  ±129 | ns |
| siloxane C | 14.17 | 267 | 5 ^a^  ±19 | - | 3 ^a^  ±7 | 180 ^b^  ±81 | 178 ^b^  ±309 | - | 2 ^a^  ±2 | <0.001^A^ |
| decamethyl-cyclopentasiloxane | 14.96 | 267 | 351  ±831 | - | 28  ±88 | - | 4002  ±6932 | - | - | ns |
| dimethoxydimethyl- silane | 16.37 | 105 | 254  ±609 | - | 261 ±768 | - | - | 45  ±100 | 144  ±162 | ns |
| siloxane D | 19.6 | 207 | 8  ±29 | - | 4  ±12 | - | - | - | 101  ±114 | ns |
| dodecamethyl- pentasiloxane | 23.82 | 147 | 3  ±11 | - | 99  ±231 | - | - | 46  ±96 | 235  ±264 | ns |
| **Terpenoids** |  |  |  |  |  |  |  |  |  |  |
| thujene | 8.429 | 93 | 173 ^a c^  ±585 | 1440 ^b^  ±861 | 210 ^a c^  ±317 | 1672 ^a c^  ±932 | 1729 ^b^  ±1062 | 347 ^a c^  ±353 | 92 ^a c^  ±104 | <0.001^A^ |
| beta-pinene | 10.06 | 93 | 201  ±771 | 312  ±334 | 585  ±830 | 1582  ±106 | 1444  ±575 | - | 234  ±264 | ns |
| 3-carene | 11.1 | 93 | 179  ±242 | 292  ±163 | 141  ±213 | 356  ±71 | 368  ±297 | 440  ±715 | 159  ±179 | ns |
| d-limonene | 11.86 | 136 | 111  ±142 | 149  ±108 | 596  ±1117 | 520  ±67 | 1038  ±848 | - | - | ns |
| gamma terpinene | 12.94 | 93 | - | 64 ^ac^  ±75 | 6 ^a^  ±13 | 188 ^b c^  ±2 | 222 ^a b c^  ±76 | - | 33 ^a^  ±37 | <0.001^B^ |
| p-cymene | 12.43 | 119 | 48 ^a^  ±149 | 265 ^a b^  ±268 | 906 ^b^  ±933 | 865 ^b^  ±322 | 1117 ^b^  ±250 | - | - | <0.001^A^ |
| eucalyptol | 12.49 | 108 | 2 ^a^  ±9 | 9 ^a c^  ±10 | 408 ^b c^  ±516 | 142 ^b^  ±21 | 160 ^b^  ±3 | 64 ^a c^  ±142 | - | <0.001^B^ |
| unknown sesquiterpenoid | 22.4 | 161 | - | - | - | 48 ^a^  ±2 | 129 ^a^  ±114 | - | - | <0.001^B^ |
| **Others** |  |  |  |  |  |  |  |  |  |  |
| **Acrylate** |  |  |  |  |  |  |  |  |  |  |
| 2-ethylhexyl acrylate | 19.05 | 55 | - | - | 4  ±11 | - | 258  ±447 | - | - | ns |
| **Chlorination byproduct** |  |  |  |  |  |  |  |  |  |  |
| ***chloroform**** | 2.66 | 83 | - | - | 3549  ±5865 | - | - | 378  ±846 | 341  ±383 | ns |
| bromodichloromethane | 4.06 | 83 | - | - | 649  ±1540 | - | - | 983  ±2197 | 255  ±193 | ns |
| bromochloronitromethane | 6.58 | 129 | - | - | 84  ±265 | - | - | 85  ±189 | 263  ±259 | ns |
| **Ether** |  |  |  |  |  |  |  |  |  |  |
| 2-ethoxy-2-methylpropane | 2.26 | 59 | 21959  ±44068 | - | 750  ±864 | - | - | - | 89  ±100 | ns |
| **Microbial metabolite** |  |  |  |  |  |  |  |  |  |  |
| indole | 25.1 | 117 | - | - | 75  ±125 | - | - | - | - | ns |
| **Nitrogenous compound** |  |  |  |  |  |  |  |  |  |  |
| N-(but-2-enoyl)butanamide | 1.95 | 69 | 2173  ±4695 | - | 16  ±41 | - | - | 76974  ±163490 | 693  ±780 | ns |
| ***acetonitrile**** | 2.17 | 41 | 3010 ^a^  ±7349 | 791 ^a^  ±1101 | 3052 ^a^  ±5344 | - | - | 41583 ^b^  ±25763 | 6027 ^a^  ±5815 | <0.001^A^ |
| **Pharmaceutical compound** |  |  |  |  |  |  |  |  |  |  |
| chlorobutanol | 14.09 | 59 | 575  ±1368 | - | 267  ±843 | 77  ±7 | 85  ±19 | - | - | ns |
| **Phenolic compound** |  |  |  |  |  |  |  |  |  |  |
| phenol | 17.08 | 94 | 0  ±1 | 1  ±1 | 17  ±33 | - | 207  ±359 | - | 9  ±10 | ns |
| **Sulphur compound** |  |  |  |  |  |  |  |  |  |  |
| dimethyl trilsulfide | 11.43 | 126 | - | - | - | - | - | 484 ^a^  ±743 | 528 ^a^  ±573 | <0.001^B^ |
| methional | 11.66 | 48 | - | - | 55  ±125 | - | - | 938  ±2098 | 1589  ±1674 | ns |
| **Unidentified** |  |  |  |  |  |  |  |  |  |  |
| unknown m/z100 | 4.15 | 100 | 66 ^a^  ±198 | 28 ^a^  ±39 | 1387 ^b^  ±2705 | 104 ^b c^  ±5 | 43 ^a b^  ±75 | - | 4140 ^c^  ±4489 | <0.001^B^ |

Data are mean (± SD). Peak area reported is relative to internal standard. RT: Retention time; Ref Ion: Reference ion; SD: Standard deviation; PBM: Preterm breastmilk; PDBM: Pasteurised donor breastmilk; BMF: Bovine milk-based fortified breastmilk; HMF: Human-milk based fortified breastmilk; RTF: Human milk-based ready-to-feed formula; LF: Liquid Formula; PF: Powdered Formula. FDR: False discovery rate adjusted *p*-value; n: number of samples analysed; Annotations represent post hoc comparisons where groups with same letter are not significantly different at p<0.05; ^A^: ANOVA/Tukey’s post hoc; ^B^: Kruskal-Wallis/Conover’s post hoc; ns: not significant; ****Identification confirmed with authentic standard***.
