# Supplementary material for: Olfactory Cues in Infant Feeds: Volatile Profiles of Different Milks Fed to Preterm Infants
Source: Front Nutr. 2021 Jan 15;7:603090. doi: 10.3389/fnut.2020.603090 (PMC7843498; doi:10.3389/fnut.2020.603090)
Supplement: Supplementary file 2 [file Table_2.docx]

**Supplementary Table 2** – Odor thresholds, odor activity value (OAV) and odor attributes of volatile compounds reported in the literature.

| Volatile compound | Odor threshold (μg L^−1^) | OAV | Matrix | Odor attributes | Reference |
| --- | --- | --- | --- | --- | --- |
| hexanal | 88 | <1 – 2 | Breastmilk | Grassy, green | 1 |
| octanal | 25 | <1 | Breastmilk | Soapy | 1 |
| nonanal | 174 | <1 | Breastmilk | Citrusy | 1 |
| decanal | 865 | <1 | Breastmilk | Fatty, soapy | 1 |
| (e)-hex-2-enal | 671 | <1 | Breastmilk | Green, grassy | 1 |
| 3-methyl butanal | 1.2 | - | Water | Malty | 2 |
| benzaldehyde | 0.3-4.6 | - | Water | - | 3 |
| butanoic acid | 4800 | 1.4 – 3.5 | Breastmilk | Sweaty | 1, 4 |
| hexanoic acid | 14,000 | <1 | Breastmilk | Musty, pungent | 1, 4 |
| octanoic acid | 22,000 | <1 | Breastmilk | Musty, plastic-like | 1, 4 |
| decanoic acid | 27,000 | <1 | Breastmilk | Fatty, rancid | 1, 4 |
| dodecanoic acid | 108,000 | <1 | Breastmilk | Fatty, rancid | 1, 4 |
| methyl octanoate | 0.2-0.9 |  | Water | - | 4 |
| methyl decanoate | 0.0043 - 0.009 | - | Water | Fruity | 4, 5 |
| ethanol | 990,000 | - | Water | Alcoholic, ethanol, pungent, sweet | 2, 6 |
| toluene | 9 | - | Milk chocolate | - | 3 |
| limonene | 6.5 | - | Whole milk | - | 3 |
| (r)-α-pinene | 4.6 | - | Water | Rosiny, fir needle-like | 2 |
| acetone | 16 | - | Milk chocolate | - | 3 |
| oct-1-en-3-one | 0.1 | <1 – 47 | Breastmilk | Mushroom-like | 1 |
| 2-heptanone | 62-98 | - | Sunflower oil | Cheese, cured ham, fruity, toasted | 3, 5 |
| pentane | 340 | - | Mineral oil |  | 3 |
| o-cymene | 0.004-0.005 | - | Air |  | 3 |

1. Spitzer J, Buettner A. Monitoring aroma changes during human milk storage at −19°C by quantification experiments. *Food Res Int* (2013) **51**:250–256. doi:10.1016/j.foodres.2012.12.002
2. Czerny M, Brueckner R, Kirchhoff E, Schmitt R, Buettner A. The influence of molecular structure on odor qualities and odor detection thresholds of volatile alkylated phenols. *Chem Senses* (2011) **36**:539–553. doi:10.1093/chemse/bjr009
3. van Gemert LJ. Odour Thresholds - Compilations of Odour threshold values in air, water and other media (second enlarged and revised edition). The Netherlands (2011). Available at: www.thresholdcompilation.com
4. Buettner A. A selective and sensitive approach to characterize Odour-active and volatile constituents in small-scale human milk samples. *Flavour Fragr J* (2007) **22**:465–473. doi:10.1002/ffj.1822.
5. Liu SQ, Holland R, Crow VL. Esters and their biosynthesis in fermented dairy products: A review. *Int Dairy J* (2004) **14**:923–945. doi:10.1016/j.idairyj.2004.02.010
6. Jia H, Chen W-L, Qi X-Y, Su M-Y. The stability of milk-based infant formulas during accelerated storage. *CyTA - J Food* (2019) **17**:96–104. doi:10.1080/19476337.2018.1561519.
